# Corticotropin releasing factor-1 receptor antagonism associated with favorable outcomes of male reproductive health biochemical parameters

**DOI:** 10.3389/fendo.2023.1127558

**Published:** 2023-05-22

**Authors:** Ahmed Khattab, R. Will Charlton

**Affiliations:** ^1^ Division of Pediatric Endocrinology, Rutgers-Robert Wood Johnson Medical School, Child Health Institute of New Jersey, New Brunswick, NJ, United States; ^2^ Spruce Biosciences, Clinical Science, Inc., South San Francisco, CA, United States

**Keywords:** congenital adrenal hyperplasia, tildacerfont, corticotropin releasing factor 1 receptor antagonists, CRF-1R, androstenedione, androstenedione/testosterone ratio

## Abstract

**Background:**

Disruption in androgen profiles and testicular adrenal rest tumors in males with congenital adrenal hyperplasia (CAH) can negatively affect sexual activity and fertility. Adrenal hyperandrogenism suppresses gonadotropin secretion and testicular adrenal rest tumors (TARTS), despite being noncancerous lesions, cause obstructive azoospermia and impaired testosterone (T) production. Circulating T in men with uncontrolled CAH is often predominantly adrenal in origin, which is reflected in high androstenedione/testosterone ratios (A4/T). Therefore, decreased luteinizing hormone (LH) levels and an increased A4/T are markers of impaired fertility in these individuals.

**Methods:**

Oral tildacerfont 200 to 1000 mg once daily (QD) (n=10) or 100 to 200 mg twice daily (n=9 and 7) for 2 weeks (Study 201), and 400 mg QD (n=11) for 12 weeks (Study 202). Outcomes measured changes from baseline in A4, T, A4/T, and LH.

**Results:**

Mean T levels increased in Study 201 from 375.5 ng/dL to 390.5 ng/dL at week 2 (n=9), 485.4 ng/dL at week 4 (n=4) and 420.7 ng/dL at week 6 (n=4). In Study 202, T levels fluctuated in the normal range from 448.4 ng/dL at baseline to 412.0 ng/dL at week 12. Mean LH levels increased in Study 201 from 0.68 IU/L to 1.59 IU/L at week 2 (n=10), 1.62 IU/L at week 4 (n=5) and 0.85 IU/L at week 6 (n=4). In Study 202, mean LH levels increased from 0.44 IU/L at baseline to 0.87 IU/L at week 12. Mean A4/T decreased across both studies. In Study 201, the mean A4/T changed from a baseline of 1.28 to 0.59 at week 2 (n=9), 0.87 at week 4 (n=4), and 1.03 at week 6 (n=4). In Study 202, the A4/T decreased from baseline of 2.44 to 0.68 at week 12. Four men were hypogonadal at baseline; all experienced improved A4/T and 3/4 (75%) reached levels <1.

**Conclusion:**

Tildacerfont treatment demonstrated clinically meaningful reductions in A4 levels, and A4/T with concomitant increased LH levels indicating increased testicular T production. The data suggests improvement in hypothalamic-pituitary-gonadal axis function, but more data is required to confirm favorable male reproductive health outcomes.

## Introduction

Classic congenital adrenal hyperplasia (CAH) due to impairment of 21-hydroxylase enzyme activity causes dysregulation of the hypothalamic-pituitary-adrenal (HPA) axis that results in cortisol deficiency and hyperandrogenemia. Defective cortisol synthesis impairs negative feedback on the hypothalamus and pituitary that normally modulates production of corticotropin releasing factor (CRF) and adrenocorticotropic hormone (ACTH). Consequently, both are increased, and excess ACTH leads to overstimulation and hyperplasia of the adrenal glands. 21-hydoxylase deficiency (21OHD) causes blockage of the glucocorticoid (GC) synthesis pathway and shunting of steroidogenesis towards the intact androgen pathway. Individuals with more severe 21OHD also have impaired mineralocorticoid synthesis and may manifest salt wasting in addition to hyperandrogenemia (1–3).

Cortisol and aldosterone deficiency can be replaced by GC and mineralocorticoid therapy at approximately physiologic doses. However, control of hyperandrogenemia often requires supraphysiological doses of GCs to adequately suppress ACTH. As a result, CAH management involves a delicate balance between risks associated with hyperandrogenemia and those associated with chronic GC overexposure.

In children, the clinical spectrum of CAH that is associated with adrenal androgen excess includes virilization of external genitalia in females, precocious adrenarche and pubarche, rapid somatic growth and accelerated skeletal maturation in early childhood which results in adult height compromise. In adults, elevated androgens are associated with insulin resistance/metabolic syndrome, acne, female hirsutism and androgenic alopecia, and reproductive concerns in men and women. Women may experience irregular menses and anovulation (1–4).

In men, disrupted androgen profiles and the relatively high prevalence of testicular adrenal rest tumors (TARTs) can affect sexual activity and fertility. Testosterone (T) levels may be normal in men with CAH, but it is often predominantly adrenal in origin, which is reflected in high androstenedione (A4) to T (A4/T) ratios. Testicular function can be impaired by suppression of the hypothalamic-pituitary-gonadal (HPG) axis due to adrenal hyperandrogenism or exogenous GC. Additionally, despite being noncancerous lesions, TARTs can impair T production and cause obstructive azoospermia (5). As a result, levels of luteinizing hormone (LH) and increased A4/T ratio are markers of impaired fertility in these individuals (6–9).

Supraphysiological GC administration, the current standard pharmacotherapy for CAH, aims to suppress the ACTH-driven adrenal hyperandrogenemia with biochemical therapeutic targets that are still higher than the normal reference ranges. In fact, normalizing 17-hydroxyprogesterone (17-OHP) levels in CAH management is likely indicative of overtreatment (2, 6, 10).

When treating patients with CAH, endocrinologists try to maintain the balance of achieving androgen control while minimizing GC overexposure. This balance is difficult to reach and most CAH patients are inevitably subjected to lifelong supraphysiological GC regimens that do not mimic typical cortisol profiles (6, 10). Supraphysiological GC levels and HPA axis dysfunction in CAH have been associated with growth failure, deranged carbohydrate and lipid metabolism, increase body mass index (BMI), decreased bone mineral density, increased cardiovascular risk, psychological morbidity and an overall impairment in quality of life (2, 10).

Due to the risks and challenges associated with the current standard of care CAH treatment, new therapies are needed. CRF1 antagonists originally became drug development targets after showing promise in animal models of anxiety, depression, and addiction. While similar studies in humans have not been successful, Schwandt and colleagues showed that CRF1 antagonism could dampen the HPA axis in humans by inhibiting CRF-mediated ACTH and cortisol release (11–14). Consequently, CRF1 antagonism became a potential target to address HPA axis dysfunction in CAH, a major unmet need. Two CRF1 receptor antagonists, tildacerfont and crinecerfont, are currently in late-stage development for CAH (10, 15, 16).

CRF1 antagonism has been shown to clinically reduce ACTH and adrenal androgen levels (17), potentially allowing the treatment goals of CAH – cortisol replacement and androgen control- to be addressed separately. Theoretically, controlling adrenal androgens by blocking CRF-mediated ACTH production should allow for cortisol replacement with physiologic doses, decreasing the compounding risk of lifelong overexposure to GCs.

This study examines changes in androgen profiles in men exposed to tildacerfont, a non-steroidal, once-daily oral CRF1 receptor antagonist, with the goal of gaining understanding of how CRF1 antagonism in men with CAH may impact gonadal Leydig cell function. The presented data highlights efficacy in suppression of adrenal hyperandrogenism and effect on TARTs (16). Novel preliminary data shows changes in gonadotropins and A4/T ratios, which may be related to positive male reproductive outcomes.

## Methods

### Ethics

Both studies were conducted in accordance with International Council for Harmonization Good Clinical Practice guidelines and the Declaration of Helsinki principals and applicable local and federal regulations. Institutional review boards at each study site approved the protocols and informed consent forms, and all participants provided written consent.

### Study design

Study SPR001-201 was a Phase 2, open-label, first-in-CAH-patients, proof-of-concept, dose-ranging study that evaluated the safety, pharmacokinetics (PK), and efficacy of repeated doses of tildacerfont in adults with classic CAH at multiple sites in the US. The study population consisted of subjects with CAH who were not adequately controlled (based on elevated 17-OHP) despite a stable GC regimen. The study consisted of 3 cohorts. Subjects in Cohort A were enrolled into a 6-week dose-escalation treatment period to identify a range of safe and effective QD doses. Subjects in Cohort A (n=10 dosed) were each treated for 2 weeks at 200 mg QD, then 2 weeks at 600 mg QD, and then 2 weeks at 1000 mg QD, with no washout between dose escalations. Subjects in Cohort B (n=9 dosed) were treated for 2 weeks at 200 mg BID. Subjects in Cohort C (n=7 dosed) were treated for 2 weeks at 100 mg BID. Subjects were enrolled sequentially into each cohort, and each cohort was completed before the start of the next cohort.

Study SPR001-202 was a Phase 2, open-label study that evaluated the safety and efficacy of tildacerfont 400 mg QD over 12 weeks of dosing in adults with classic CAH. Subjects who previously participated in Study SPR001-201 were eligible to enroll in this study (after a washout period of at least 45 days), along with tildacerfont-naïve subjects. Nine of the 11 subjects enrolled were rollover subjects from Study SPR001-201.

During the studies, participants continued their previously prescribed regimen of GC ± mineralocorticoid replacement without dose adjustments.

Primary analyses have been published (16) but this *post-hoc* analysis only includes data from male participants, as it focuses on male reproductive health by evaluating changes in A4, T, LH, A4/T ratio, and ultrasound measurement of TARTs.

Study 201 included visits at baseline (Day 1) and week 2 for all cohorts, as well as weeks 4 and 6 for Cohort A dose escalation. Study 202 included visits at baseline (Day 1) and weeks 2, 4, 6, 8, 10, and 12. Biomarkers were drawn at 8am prior to administration of the morning GC dose. Study 202 participants had a follow-up visit 30 days after study drug discontinuation.

### Statistical analyses

Sample size for Study 201 was estimated based on the assumption that 6-9 participants per cohort would provide adequate initial safety data and support proof of concept. For Study 202, estimates were based on the number of participants expected to enroll after completing Study 201. Power calculations were not performed, and all statistics are descriptive in nature.

The safety analysis population included all participants who received at least one dose of tildacerfont. The pharmacokinetic analysis population included all participants who had evaluable PK profiles. The efficacy analysis population included all participants who had both baseline and post-baseline 8am biomarker measurements. Due to the dynamic range of these biomarkers, and the nonnormality of the data, geometric means and geometric mean percentage changes were used to summarize the changes over time.

## Results

10 men enrolled in Study 201: 5 in Cohort A, 1 in Cohort B, and 4 in Cohort C. 4 men enrolled in Study 202, 3 of whom had participated in Study 201. Due to the washout period between studies, results are evaluated by measurement instead of individual (i.e., measurements from 201 participants who enrolled in 202 were treated as new data). Also, while Study 202 results are presented as changes from baseline to post-treatment (week 12), results from Study 201 are presented at weeks 2, 4 and 6 due to dose increases.

Mean age of the men in both studies combined was 35.1 years (range 18-54): 46.3 years in Study 201 and 32.3 years in Study 202. All participants reported race as white except one who identified as white and Asian. Four reported ethnicity as Hispanic or Latino.

All the participants had elevated 17OHP at baseline, and all but one had elevated ACTH and A4 levels as well. One participant had modest elevation of baseline 17OHP with normal ACTH and A4 levels and normal A4/T ratio at 0.14. Aside from this individual and one non-responder, decreased ACTH levels resulted in altered androgen profiles across both studies.

At baseline, most of the men had normal T levels, but four 201 participants had T<300ng/dL. 6/11 (55%) had suppressed baseline LH (including the four with low T), and 11/14 (79%) had A4/T ratios > 1, indicating a predominantly adrenal origin of T.

During treatment with tildacerfont, mean T levels increased in Study 201 from 375.5 ng/dL to 390.5 ng/dL at week 2 (n=9), 485.4 ng/dL at week 4 (n=4) and 420.7 ng/dL at week 6 (n=4). In Study 202, mean T levels fluctuated within the normal range from 448.4 ng/dL at baseline to 412.0 ng/dL at week 12.

Mean LH levels increased in Study 201 from 0.68 IU/L to 1.59 IU/L at week 2 (n=10), 1.62 IU/L at week 4 (n=5) and 0.85 IU/L at week 6 (n=4). In Study 202, mean LH levels increased from 0.44 IU/L at baseline to 0.87 IU/L at week 12. Of the four men who had low T and suppressed LH (hypogonadotropic hypogonadism), all showed improvements in LH and 3/4 (75%) normalized LH levels.

Mean A4/T ratios decreased across both studies. In Study 201, the mean changed from a baseline of 1.28 to 0.59 at week 2 (n=9), 0.87 at week 4 (n=4), and 1.03 at week 6 (n=4). In Study 202, the A4/T ratio decreased from baseline of 2.44 to 0.68 at week 12. All the hypogonadal men experienced improved A4/T ratios, and 3/4 (75%) reached levels <1.

Three men had evaluable pre- and post-treatment scrotal ultrasounds to assess TARTs. In Study 201, one participant in Cohort A experienced a 23% reduction in TART volume after 6 weeks of treatment, correlated with an 87% reduction in ACTH. In Study 202, the non-responder showed no change in TART volume, correlated with no change in ACTH levels. Another 202 participant had 4 TARTs at baseline that were undetectable after treatment. However, assessment is confounded due to increased dexamethasone exposure during the study.

## Discussion

Males with CAH may experience primary hypogonadism due to testicular damage caused by TARTs or secondary hypogonadism (hypogonadotropic hypogonadism) due to suppression of LH by adrenal T as well as adrenal androgens that have been aromatized to estrone or estradiol. Normal T levels with suppressed gonadotropins and high A4 levels suggest a higher contribution of adrenal T to circulating T levels.

In a cross-sectional clinical outcome study involving 14 study centers in 6 European countries, Engels et al. evaluated a cohort of 121 men with CAH and extrapolated that approximately 20% of men with CAH have low T, and around half of those have suppressed gonadotropins. Our findings, albeit in a small cohort, included 29% (4/14) with low T. Interestingly, all four of these men had suppressed LH levels.

In a prospective longitudinal monocentric study of sexual well-being that included 20 male patients with CAH, serum A4/T ratio was used as a biomarker to differentiate testicular vs. adrenal T production. An A4/T of <0.2 indicated normal testicular T production, whereas an A4/T of >1 suggested T was predominately of adrenal origin. Auchus and Arlt believe that an A4/T ratio greater than 0.5 is an indicator of significant adrenal contribution to the total T level in males with CAH and if the A4/T ratio is greater than 1 with concomitant LH suppression then the majority of the circulating T is of adrenal origin (17, 18).

Our data demonstrates clinically meaningful decrease in A4 levels, increase in the testicular T proportion, and increase LH levels, collectively suggestive of recovery HPG axis function in males with CAH receiving tildacerfont. **Table 1** shows the decrease in A4/T ratios with concomitant increase in LH levels in SPR001-201. **Table 2** shows the decrease in A4 levels throughout the course of the 12 weeks of tildacerfont 400 mg daily administration and concomitant increase in LH levels in SPR001-202. Despite a mean decrease in T, levels were consistently normal and changes in the A4/T ratio suggest an increase in the testicular contribution. These results are consistent with clinical trial data reported from the CRF1 receptor inhibitor crinecerfont (9).

**Table 1:** Results from SPR001-201 showing changes in serum A4, Testosterone, LH, and A4/T ratio.

Laboratory Parameter		Baseline	Week 2	Week 4	Week 6
Androstenedione (ng/dL)	n	10	10	4	4
	Geometric Mean (CV%)	459.7 (143.8)	223.5 (304.6)	420.7 (428.5)	429.4 (411.1)
	Geometric Mean % change	n/a	-51.4%	-22.9%	-21.3%
Testosterone (ng/dL)	n	9	9	4	4
	Geometric Mean (CV%)	375.52 (63.8)	390.48 (40.7)	485.40 (60.8)	420.71 (72.0)
	Geometric Mean % change	n/a	+4.0%	+23.6%	+7.2%
Androstenedione: Testosterone	n	9	9	4	4
(Ratio)	Geometric Mean (CV%)	1.283 (193.9)	0.594 (594.9)	0.867 (960.8)	1.030 (1144.1)
	Geometric Mean % change	n/a	-53.7%	-37.3%	-25.5%
Luteinizing Hormone (IU/L)	n	10	10	5	4
	Geometric Mean (CV%)	0.680 (258.7)	1.585 (221.0)	1.619 (253.3)	0.851 (740.9)
	Geometric Mean % change	n/a	+133.1%	+232.1%	+30.0%

CI, confidence interval; CV, coefficient of variance.

**Table 2:** Results from SPR001-202 showing changes in serum A4, Testosterone, LH, and A4/T ratio.

Laboratory Parameter		Baseline	Week 2	Week 4	Week 6	Week 8	Week 10	Week 12	Follow-up
			TILDACERFONT	400 mg	PO QD				
	n	4	4	4	4	4	4	4	4
Androstenedione (ng/dL)	Geometric Mean (CV%)	1096.5 (46.1)	437.8 (312.6)	427.3 (448.1)	338.6 (241.2)	419.5 (258.4)	256.4 (371.6)	281.2 (345.2)	591.6 (252.7)
	Geometric Mean % change	n/a	-60.1%	-61.0%	-69.1%	-61.7%	-76.6%	-74.3%	-46.0%
Testosterone (ng/dL)	Geometric Mean (CV%)	448.44 (26.7)	348.90 (109.2)	395.82 (118.6)	306.03 (116.1)	368.01 (115.7)	422.45 (127.9)	412.01 (127.5)	447.79 (34.9)
	Geometric Mean % change	n/a	-22.2%	-11.7%	-31.8%	-17.9%	-5.8%	-8.1%	-0.1%
Androstenedione: Testosterone (Ratio)	Geometric Mean (CV%)	2,444 (62.0)	1.253 (714.5)	1.078 (813.7)	1.094 (799.7)	1.139 (589.3)	0.602 (1014.9)	0.679 (1033.2)	1.310 (446.5)
	Geometric Mean % change	n/a	-48.7%	-55.9%	-55.3%	-53.4%	-75.4%	-72.2%	-46.4%
Luteinizing Hormone (IU/L	Geometric Mean (CV%)	0.436 (316.4)	0.545 (386.1)	0.653 (475.2)	0.812 (662.8)	0.667 (469.8)	0.874 (787.9)	0.866 (780.3)	0.600 (410.6)
	Geometric Mean % change	n/a	25.1%	50.0%	86.5%	53.2%	100.7%	98.8%	37.7%

CI, confidence interval; CV, coefficient of variance.

**Table 2** also shows that 30 days after discontinuing tildacerfont (follow up column) levels of A4, T, LH, and A4/T ratio all reverted towards baseline, supporting the hypothesis that the changes observed during the study were due to tildacerfont exposure.

While conclusions cannot be drawn due to small numbers of participants, it is noteworthy that all four men with baseline hypogonadotropic hypogonadism showed improvement in LH and A4/T ratios, and three of them normalized LH levels and reached A4/T ratios <1.

This *post-hoc* analysis supports prior studies showing that HPG axis disruption is common in men with CAH and suggests that modulation of adrenal androgen production through CRF1 antagonism may improve gonadal Leydig cell function. This analysis is primarily evaluating Leydig cell function using LH, T and A4/T rather than Sertoli cell function, as specific markers of Sertoli cell function such as inhibin levels or semen analysis, were not analyzed.

A potential pitfall is that the use of total testosterone rather than free testosterone may not accurately reflect hyperandrogenic states associated with low sex hormone binding globulin levels in individuals with a high BMI.

## Data availability statement

The raw data supporting the conclusions of this article will be made available by the authors, without undue reservation.

## Ethics statement

Both studies were conducted in accordance with International Council for Harmonization Good Clinical Practice guidelines and the Declaration of Helsinki principals and applicable local and federal regulations. Institutional review boards at each study site approved the protocols and informed consent forms, and all participants provided written consent. The patients/participants provided their written informed consent to participate in this study.

## Author contributions

All authors listed have made a substantial, direct, and intellectual contribution to the work and approved it for publication. Both authors contributed to data analysis and writing of the manuscript.

## References

[1] El-Maouche D Arlt W Merke DP. Congenital adrenal hyperplasia. Lancet (2017) 390(10108):2194–210. doi: 10.1016/S0140-6736(17)31431-9 28576284

[2] Khattab A Marshall I. Management of congenital adrenal hyperplasia: beyond conventional glucocorticoid therapy. Curr Opin Pediatr (2019) 31 (4) :550–4. doi: 10.1097/MOP.0000000000000780 31295195

[3] Ng SM Stepien KM Krishan A. Glucocorticoid replacement regimens for treating congenital adrenal hyperplasia. Cochrane Database Syst Rev (2020) 3:CD012517. doi: 10.1002/14651858.CD012517.pub2 32190901 PMC7081382

[4] Lin-Su K Harbison MD Lekarev O Vogiatzi MG New MI. Final adult height in children with congenital adrenal hyperplasia treated with growth hormone. J Clin Endocrinol Metab (2011) 96(6):1710–7. doi: 10.1210/jc.2010-2699 PMC3206397 21450983

[5] Kim MS Goodarzian F Keenan MF Geffner ME Koppin CM De Filippo RE. Testicular adrenal rest tumors in boys and young adults with congenital adrenal hyperplasia. J Urol (2017) 197(3 Pt 2):931–6. doi: 10.1016/j.juro.2016.09.072 PMC5449264 27840017

[6] Speiser PW Arlt W Auchus RJ Baskin LS Conway GS Merke DP. Congenital adrenal hyperplasia due to steroid 21-hydroxylase deficiency: An endocrine society clinical practice guideline. J Clin Endocrinol Metab (2018) 103(11):4043–88. doi: 10.1210/jc.2018-01865 PMC6456929 30272171

[7] King TF Lee MC Williamson EE Conway GS. Experience in optimizing fertility outcomes in men with congenital adrenal hyperplasia due to 21 hydroxylase deficiency. Clin Endocrinol (Oxf) (2016) 84(6):830–6. doi: 10.1111/cen.13001 26666213

[8] Falhammar H Nystrom HF Ekstrom U Granberg S Wedell A Thoren M. Fertility, sexuality and testicular adrenal rest tumors in adult males with congenital adrenal hyperplasia. Eur J Endocrinol (2012) 166(3):441–9. doi: 10.1530/EJE-11-0828 PMC3290120 22157069

[9] Auchus RJ Sarafoglou K Fechner PY Vogiatzi MG Imel EA Davis SM. Crinecerfont lowers elevated hormone markers in adults with 21-hydroxylase deficiency congenital adrenal hyperplasia. J Clin Endocrinol Metab (2022) 107(3):801–12. doi: 10.1210/clinem/dgab749 PMC8851935 34653252

[10] Merke DP Mallappa A Arlt W Brac de la Perriere A Linden Hirschberg A Juul A. Modified-release hydrocortisone in congenital adrenal hyperplasia. J Clin Endocrinol Metab (2021) 106(5):e2063–e77. doi: 10.1210/clinem/dgab051 PMC8063257 33527139

[11] Binneman B Feltner D Kolluri S Shi Y Qiu R Stiger T. A 6-week randomized, placebo-controlled trial of CP-316,311 (a selective CRH1 antagonist) in the treatment of major depression. Am J Psychiatry (2008) 165(5):617–20. doi: 10.1176/appi.ajp.2008.07071199 18413705

[12] Coric V Feldman HH Oren DA Shekhar A Pultz J Dockens RC. Multicenter, randomized, double-blind, active comparator and placebo-controlled trial of a corticotropin-releasing factor receptor-1 antagonist in generalized anxiety disorder. Depress Anxiety (2010) 27(5):417–25. doi: 10.1002/da.20695 20455246

[13] Kwako LE Spagnolo PA Schwandt ML Thorsell A George DT Momenan R. The corticotropin releasing hormone-1 (CRH1) receptor antagonist pexacerfont in alcohol dependence: a randomized controlled experimental medicine study. Neuropsychopharmacology (2015) 40(5):1053–63. doi: 10.1038/npp.2014.306 PMC4367465 25409596

[14] Schwandt ML Cortes CR Kwako LE George DT Momenan R Sinha R. The CRF1 antagonist verucerfont in anxious alcohol-dependent women: Translation of neuroendocrine, but not of anti-craving effects. Neuropsychopharmacology (2016) 41(12):2818–29. doi: 10.1038/npp.2016.61 PMC5061889 27109623

[15] Prete A Auchus RJ Ross RJ. Clinical advances in the pharmacotherapy of congenital adrenal hyperplasia. Eur J Endocrinol (2021) 186(1):R1–R14. doi: 10.1530/EJE-21-0794 34735372 PMC8679847

[16] Sarafoglou K Barnes CN Huang M Imel EA Madu IJ Merke DP. Tildacerfont in adults with classic congenital adrenal hyperplasia: Results from two phase 2 studies. J Clin Endocrinol Metab (2021) 106(11):e4666–e79. doi: 10.1210/clinem/dgab438 PMC8530725 34146101

[17] Verhees MJM Engels M Span PN Sweep F van Herwaarden AE Falhammar H. Quality of life in men with congenital adrenal hyperplasia due to 21-hydroxylase deficiency. Front Endocrinol (Lausanne) (2021) 12:626646. doi: 10.3389/fendo.2021.626646 33815285 PMC8018222

[18] Auchus RJ Arlt W. Approach to the patient: the adult with congenital adrenal hyperplasia. J Clin Endocrinol Metab (2013) 98(7):2645–55. doi: 10.1210/jc.2013-1440 PMC3701266 23837188

